# Coagulation Disorders in Subjects Undergoing Pump-Driven Veno-Venous Ecco2-R For Severe Acute Hypercapnic Respiratory Failure - a Single Center Experience

**DOI:** 10.1186/2197-425X-3-S1-A512

**Published:** 2015-10-01

**Authors:** U Harler, GF Lehner, J Hasslacher, M Joannidis

**Affiliations:** Medical University of Innsbruck, Division of Intensive Care and Emergency Medicine, Department of Internal Medicine, Innsbruck, Austria

## Introduction

Recent evidence suggests low-flow extracorporeal CO2 removal (ECCO2-R) systems as safe and promising adjunctive therapy to avoid endotracheal intubation and the related negative consequences in subjects with severe hypercapnic respiratory failure [1]. in high-flow extracorporeal membrane oxygenation systems heterogeneous coagulation disorders are a well-known complication. However, to date there is little evidence for the influence of pump-driven low-flow veno-venous ECCO2-R on the coagulation system.

## Objectives

This study is a retrospective analysis of four subjects developing coagulation disorders with bleeding complications while undergoing ECCO2-R.

## Methods

Four subjects treated with a pump-driven veno-venous ECCO2-R (system: iLA Activve®; membrane ventilator: Minilung®; Novalung GmbH, Talheim, Germany) for severe hypercapnic respiratory failure due to acute exacerbation of COPD were included in this study. Unfractionated heparin was used for anticoagulation with a target aPTT of 45-55 sec. Coagulation parameters i.e. hemoglobin, platelets, fibrinogen, antithrombin and D-DIMER were retrieved from the charts at treatment initiation and during the time range starting 72 hours before and ending at the clinical onset of the bleeding complication.

## Results

Mean application time of ECCO2-R was 196.5 h ( ± 77.4) with an average blood flow of 1.1 l/min ( ± 0.2). Bleeding events consisted of two pulmonary bleedings, one large soft tissue hematoma and one hemothorax. Coagulation parameters are depicted below in Table 1. ECCO2-R was removed in all subjects after onset of the bleeding complication resulting in stabilization of the coagulation state.

**Table 1:** *Coagulation parameters.*

	baseline*	-72h	-48h	-24h	day of bleeding
**hemoglobin (G/l)**	114.5 ( ± 24.3)	97.8 ( ± 11.8)	88.8 ( ± 16.9)	79 ( ± 14.5)	81.8 ( ± 14.1)
**thrombocytes (G/l)**	195.5 ( ± 125.5)	193.3 ( ± 136.0)	171 ( ± 122.5)	141.8 ( ± 122.1)	125.5 ( ± 100.2)
**fibrinogen (mg/dl)**	370 ( ± 97.4)	358.8 ( ± 133.9)	343.5 ( ± 136.3)	255.5 ( ± 136.2)	235.5 ( ± 142.9)
**AT III (%)**	101 ( ± 20.9)	86 ( ± 25.7)	80.5 ( ± 21.6)	69.5 ( ± 23.7)	74 ( ± 14.5)
**D-DIMER (µg/l)**	1170 ( ± 435.4)	5079 ( ± 6597)	7569 ( ± 11340)	11048 ( ± 16140)	12709 ( ± 15453)
**PT (%)**	90.8 ( ± 18.7)	91 ( ± 19.8)	86.8 ( ± 18.8)	81.8 ( ± 16.9)	82.5 ( ± 24.8)
**aPTT (sec)**	38.8 ( ± 12.0)	39.8 ( ± 10.4)	46.8 ( ± 11.3)	49.5 ( ± 15.1)	36.8 ( ± 7.7)
**Heparin (IU/kg/day)**	0	207.8 ( ± 80.5)	259.7 ( ± 84.3)	150.1 ( ± 115.7)	78.33 ( ± 97.1)

Results are presented as mean ( ± SD).

*baseline refers to the last value before application of ECCO2-R

## Conclusions

Despite adequate anticoagulation subjects undergoing pump-driven veno-venous ECCO2-R developed coagulation disorders similar to disseminated intravascular coagulation with concomitant bleeding complications. the underlying mechanism remains to be clarified.
